# Serologic Evidence of West Nile Virus Infection in Horses, Coahuila State, Mexico

**DOI:** 10.3201/eid0907.030166

**Published:** 2003-07

**Authors:** Bradley J. Blitvich, Ildefonso Fernandez-Salas, Juan F. Contreras-Cordero, Nicole L. Marlenee, Jose I. Gonzalez-Rojas, Nicholas Komar, Duane J. Gubler, Charles H. Calisher, Barry J. Beaty

**Affiliations:** * Colorado State University, Fort Collins, Colorado, USA; † Universidad Autonoma de Nuevo Leon, Nuevo Leon, Mexico; ‡ Centers for Disease Control and Prevention, Fort Collins, Colorado, USA

**Keywords:** West Nile virus, flavivirus, Mexico, horse, surveillance, dispatch

## Abstract

Serum samples were obtained from 24 horses in the State of Coahuila, Mexico, in December 2002. Antibodies to West Nile virus were detected by epitope-blocking enzyme-linked immunosorbent assay and confirmed by plaque reduction neutralization test in 15 (62.5%) horses. We report the first West Nile virus activity in northern Mexico.

West Nile virus (WNV; family *Flaviviridae*, genus *Flavivirus*) is a member of the Japanese encephalitis virus complex, which also includes Japanese encephalitis virus, Saint Louis encephalitis virus (SLEV), and Murray Valley encephalitis virus (*1*). These viruses are maintained in cycles between mosquitoes and birds (*2*). The principal vectors for WNV are *Culex* species mosquitoes, and many species of wild birds act as vertebrate hosts (*3*). Humans, horses, and other mammals usually serve as dead-end hosts. In humans and equines, WNV infection is usually asymptomatic or characterized by a mild febrile illness, although fatal meningoencephalitis or encephalitis may occur (*3*–*5*). WNV has a broad geographic distribution, recently including North America (*4*,*5*). The initial outbreak of WNV in North America was recognized in New York City in August 1999. Since then, WNV’s geographic range has increased. WNV activity has now been reported in 44 states in the United States, the District of Columbia, and 5 of the 10 Canadian provinces (*6*,*7*).

In anticipation of the possible emergence of WNV into Mexico, we conducted equine infection surveillance in the northeastern states of Mexico. Coahuila State is bordered on the north by Texas. WNV activity has been detected in 204 (80%) of 254 Texas counties, including most counties that border Coahuila State (*8*). Therefore, Coahuila State was considered to be a likely point of incursion of WNV into Mexico from the USA.

## Case Study

We present data from a small equine serosurvey conducted in Coahuila State in December 2002. A more extensive equine serosurvey is currently under way and will be described in detail elsewhere (B.J. Beaty, unpub. data). In the present study, blood samples were taken from 24 domestic horses at study sites located in Ciudad Acuña, Jiménez, and Saltillo (Figure). The Ciudad Acuña and Jiménez sites are approximately 40 km apart, and both are <15 km from the Texas border. Saltillo is located in the southeast region of Coahuila State and is approximately 220 km from Texas. All study sites are privately owned ranches.

**Figure:**
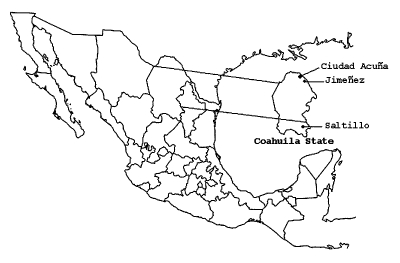
Geographic location of West Nile virus study sites in Coahuila State, Mexico.

The climatic conditions of the three study sites are similar and can be described as hot, dry, and arid. The average annual temperature ranges from 18°C to 22°C. The average rainfall is from 100 to 300 mm per year. The sites in Ciudad Acuña and Jiménez are approximately 300 m above sea level. The Saltillo site is situated at an elevation of approximately 1500 m.

A horse from the Ciudad Acuña ranch died October 17, 3 days after being observed with neurologic signs. The case was not reported immediately, and we were unable to obtain a tissue specimen postmortem. On December 19, with the assistance of a local veterinary practitioner, we sampled 14 horses at this site, 5 of which had developed neurologic disease in mid- to late October. Clinical symptoms included ataxia, weakness of limbs, trembling, and anxiety. All five horses survived. The horses with clinical signs were from 1 to 5 years of age; three were male, and two were female. Ages and sexes of horses without clinical symptoms were not documented. The veterinarian reported a great abundance of mosquitoes in the area. Another six horses were sampled in Jiménez and four in Saltillo, none of which had signs of illness. According to the owners, none of the horses had ever been outside the State of Coahuila, and none of the horses had been vaccinated against WNV.

All serum samples were tested for antibodies to WNV by epitope-blocking enzyme-linked immunosorbent assay (ELISA). Blocking ELISAs were performed by using the WNV-specific monoclonal antibody (MAb) 3.1112G, as previously described (*9*). The ability of the Mexican horse serum samples to block the binding of the MAb to WNV antigen was compared to the blocking ability of horse serum without antibody to WNV (Vector Laboratories, Burlingame, CA). Data were expressed as relative percentages by using the formula of Hall et al. (*10*). Previously, we considered an inhibition value >30% to indicate the presence of viral antibodies (*9*). Recently, we have shown that ELISAs performed with MAb 3.1112G detect WNV antibodies in various vertebrate species, including horses (*9*,*11*).

Fourteen serum samples were positive in blocking ELISA that utilized MAb 3.1112G (Table). Serum from another horse (H-16) inhibited the binding of MAb by 25%, which is close to the diagnostic criterion. Previously, we observed that the nonspecific inhibition values for serum samples from noninfected control birds ranged from 0% to 24.3% (*9*). Therefore, if we used a less stringent threshold value of >25%, this serum could be considered positive for WNV antibodies.

**Table:** Summary of serologic data for horses in Coahuila State, Mexico^a^

Horse	Study site	Clinical illness	^%^ inhibition by ELISA^b^	^c^PRNT_90_ titer		PRNT diagnosis
				WNV	SLEV	
H-1	Ciudad Acuña	No	90	>320	^—d^	WNV
H-2	Ciudad Acuña	No	5	—	—	Negative
H-3	Ciudad Acuña	No	0	—	—	Negative
H-4	Ciudad Acuña	No	0	—	—	Negative
H-5	Ciudad Acuña	No	90	>320	40	WNV
H-6	Ciudad Acuña	No	93	>320	—	WNV
H-7	Ciudad Acuña	No	93	>320	—	WNV
H-8	Ciudad Acuña	Yes	93	>320	—	WNV
H-9	Ciudad Acuña	Yes	86	>320	—	WNV
H-10	Ciudad Acuña	Yes	90	>320	20	WNV
H-11	Ciudad Acuña	No	89	>320	—	WNV
H-12	Ciudad Acuña	No	92	>320	20	WNV
H-13	Ciudad Acuña	Yes	91	>320	—	WNV
H-14	Ciudad Acuña	Yes	78	>320	20	WNV
H-15	Jiménez	No	82	>320	—	WNV
H-16	Jiménez	No	25	40	—	WNV
H-17	Jiménez	No	7	—	—	Negative
H-18	Jiménez	No	93	>320	20	WNV
H-19	Jiménez	No	0	20	20	Flavivirus
H-20	Jiménez	No	47	40	—	WNV
H-21	Saltillo	No	9	—	—	Negative
H-22	Saltillo	No	15	—	—	Negative
H-23	Saltillo	No	12	—	—	Negative
H-24	Saltillo	No	11	—	—	Negative

^a^ELISA, enzyme-linked immunosorbent assay; PRNT, plaque reduction neutralization test; WNV, West Nile virus; SLEV, Saint Louis encephalitis virus. ^b^Inhibition values >30% are considered significant. ^c^Neutralizing antibodies to WNV in selected horse serum samples were confirmed at Centers for Disease Control and Prevention-Division of Viral and Bacterial Infectious Diseases by PRNT. ^d^—, <20.

To validate the above assays, we tested serum samples for neutralizing antibodies to WNV and SLEV by plaque reduction neutralization assay (PRNT). Testing for neutralizing antibody to SLEV was important because this virus is enzootic in the Americas and antibodies to WNV and SLEV often cross-react. Furthermore, horses are susceptible to SLEV infection, although clinical manifestations have not been reported (*12*). Viral isolates of WNV (strain NY99-35261-11) and SLEV (strain TBH-28) were obtained from the World Health Organization Center for Arbovirus Reference and Research, maintained at the Centers for Disease Control and Prevention, Division of Vector-Borne Infectious Diseases, Fort Collins, CO. PRNTs were performed by using Vero cells. Serum samples were tested by using a starting dilution of 1:20. Titers were expressed as the reciprocal of serum dilutions reducing the number of plaques that were >90% (PRNT_90_).

## Conclusions

Overall, PRNT and ELISA data were in concordance. Fifteen (62.5%) horses were considered to be seropositive for WNV by PRNT because the antibody titers for WNV were less than fourfold higher that the corresponding SLEV titer (Table). These 15 were the same serum samples that had inhibition values of >25% by ELISA. Of these, 11 horses were from Ciudad Acuña, and 4 were from Jiménez. Evidence for WNV infections was detected in 5 (100%) of 5 horses with clinical symptoms, and 10 (52.6%) of 19 horses without clinical symptoms. Therefore, the rate of asymptomatic seropositivity was high, with 10 (66.7%) of 15 WNV-infected horses showing no signs of illness. Similarly, 21 (58.3%) of 36 WNV-infected horses sampled during a serosurvey in New York in 1999 showed no clinical signs (*13*). However, the sample population (n=24) in the present serosurvey was notably small, and data from our large equine serosurvey will provide a more reliable estimate of the asymptomatic seropositivity rate.

We were unable to detect RNA in any horse serum by reverse-transcription polymerase chain reaction with WNV-specific primers (*14*). We plan to isolate and amplify WNV RNA sequences from tissue specimens obtained from seropositive horses, as well as from birds, in future studies.

We are currently conducting avian infection surveillance in the State of Coahuila and the neighboring states of Tamaulipas and Nuevo Leon. Preliminary evidence suggests that several birds from a region in Nuevo Leon State have antibodies to WNV (I. Fernandez-Salas, unpub. data). The birds were trapped in February 2003, 2 months after we obtained samples from the horses in Coahuila State. However, equine cases often precede the detection of seropositive birds. For example, an equine case was the first indication of WNV activity in 29% (660/2,289) of the United States counties to report virus activity in 2002 (*6*).

In summary, we have obtained serologic evidence for antibodies to WNV in horses in the State of Coahuila, Mexico. In the accompanying manuscript, we report the detection of antibodies to WNV in horses in the State of Yucatan (*15*). These two reports provide the first published evidence of WNV activity in horses in Mexico. Antibodies to WNV, or a closely related virus, were detected in a single bovine during a serosurvey in Chiapas, Mexico, in mid-2001 (*16*). WNV will probably become endemic in Mexico, which is a major concern to public health authorities in the Americas. Our findings demonstrate the importance for continued WNV surveillance in Mexico.
